# Dr. Trotter, on Nitrous Fumigation

**Published:** 1800-04

**Authors:** T. Trotter

**Affiliations:** Hamoaze


### *To the Editors of the Medical and Phyſical Journal.*

Gentlemen,

YOU will very much oblige me by inſerting the incloſed Remarks in your next Number of the Medical and Phyſical Journal.

It is with much pleaſure that I witneſs the increaſing circulation of your publication among the Surgeons of the Fleet; for in their department it is ſingularly uſeful, by giving a compendium of all improvements, and enabling them to apply the ſame to their own practice. I am,

Gentlemen,

With much conſideration,

Your very humble ſervant,

T. TROTTER.

*Hamoaze, March 5, 1800.*

IN the 4th volume of The Annals of Medicine, (1799) Dr. Yeats, of Bedford, has made ſome remarks on fumigation with *nitrous vapour*, and pointed out a few miſtakes in my ſtatement of the ſubject. Theſe miſtakes have already produced an apology, on my part, in the 2d volume of *Medicina Nautica.*

On looking over my letter publiſhed in the laſt Number of the Medical and Phyſical Journal, I obſerve the words *nitrous vapour* uſed throughout; my error was therefore corrected, at leaſt a month, before meeting with the caſtigation of Doctor Yeats.

In thus changing the term, becauſe the degree of *oxygenation* of this vapour has not been aſcertained by its author, and becauſe *nitrous gas* implies a preciſe quantity of *vital air* in combination with *azote*, it is not to be inferred that I mean any compromiſe of opinion with either the principles or practice. The firſt I hold to be vague and undefined; and the latter is unſupported by any clear or deciſive teſtimony. When both the principles and practice ſhall be proved from indiſputable authority, it will then remain for the favorers of *fumigation* to demonſtrate, by equally indubitable proofs, the chemical nature of that power or ſubſtance, which only, for the ſake of explaining a fact, we call CONTAGION. When this is done, they will reſcue their darling *antidote* from the opprobious epithet of quackery, as being the relic of a barbarous and ſuperſtitious age.

With reſpect to what I ſaid of the combinations of *azote* with *caloric* and *oxygen*, the miſconception is ſuch as might have eaſily happened in quoting from memory. But the ſituation of Dr. Yeats, perhaps, does not enable him to make allowance for the ſhifting ſcene to which our ſtudies are expoſed. Our motto has not been, *fortiter occupa portum.*

Having now confeſſed my ſins, let me turn to what Dr. Yeats calls a "further proof," in favour of *nitrous fumigation* arreſting the progreſs of a jail fever. The moſt valuable part of the evidence, in this inſtance, is kept in the back ground. The hiſtory of the caſe is imperfect. It ought to have been mentioned, whence this contagion came; or how it was generated in the priſon; whether by foul air, damp and cold apartments, and perſonal filth joined to irregularities or depreſſing paſſions. If any of theſe cauſes produced it, I can well ſuppoſe, the penetrating eye and ſympathizing heart of the attending phyſician would quickly diſcern them, and ſee that they were ſpeedily corrected. When ſuch ſalutary precautions are taken, is it fair to overlook them, and attribute every healthy change in the condition of the patient to fumigation? I know it may be ſaid, as it has been by all the ſupporters of this Empiriciſm piricifm, that without fumigation, *cleanlinefs*, *ventilation*, *&c.* will not be fufficient. But to this we oppofe ten thoufand facts, collected on the largeft fcale, in the Channel Fleet; and more accumulating at the moment I am writing. We cannot be accufed of troubling our readers with folitary cafes of infection; yet there was fcarcely a cruize in which the Hofpital-fhip did not receive patients in typhus: but no perfon was ever infected there. It therefore becomes a folemn duty on my part, to guard officers againft confidence in any *preventive* that is in danger of attracting their attention from *means of fafety* that have received the fanction of experience, and that difclaim all myftery.

I can allow Dr. Yeats full credit for the intereft he takes in the health of his Majefty's naval fubjects, in this difcuffion; and if he looks back to the facility with which feventeen fail of the line and five frigates were cleared after the victory of the First of June, he will not be afraid to truft it to the prefent rules of practice. When fevers become prevalent in fhips, the eafe with which they are expelled is not only more certain, but they no longer appear under that malignant character, or followed by the fame number of deaths, that marked their progrefs in former times. This part of prophylactic means, without the leaft jealoufy, I freely confign to officers; and if ever they tamely yield it up to the trumpery which has lately been introduced among us, with a fuccefs that was worthy of a better caufe, they will deferve to lofe their commiffions. Men who are accuftomed to fuch matchlefs prowefs in their own profeffion, cannot be infenfible to a duty the moft effential to fecure the acquifition of their reputation and fortune.

A mind like that of Dr. Yeats, familiarized to inveftigation, with a powerful chemical agent in his hands, whofe attractions are well known, could not fail to attempt fome explanation of its manner of operation againft contagious matter. In one part of his Critique, fpeaking of the *nitric vapour*, he fays: "It will readily part with it, (oxygen) and thus render the atmofphere purer." Here then he fully gives up the point, that it *directly* attacks *contagious miasma*; and confequently, it muft act by meliorating the refpirable portion of the air. But he gives no proof that the *nitric acid*, converted into *vapour*, yields oxygen to the common atmofphere. I apprehend, if the Doctor attends to the evolution of the fumes from the pipkins, he will find no feparation of *oxygen*, unlefs it fhould be attracted by fome filthy exhalations, which have in their compofition *hydrogenous gas*, as happened in the dirty wards of the Union hofpital-fhip. If, however, Dr. Yeats means that the only end in view from fumigation, ought to be to increafe the oxygenous quality of the atmoſphere, in ſick apartments, I ſhall moſt readily ſubſcribe to the principle. But, if he admits this, he will think alſo with me, that chemiſtry, at the preſent day, affords a proceſs much ſuperior to what can be effected by the decompoſition of *nitre*, or of *ſea ſalt*, in the *muriatic acid gas*, which deſtroys unpleaſant ſmells, in the ſame manner as nitrous vapour neutralizes the foul effluvia of unpurified utenſils. In the wards of hoſpitals, the decks of ſhips, or the cells of a jail, if windows cannot effectually throw in pure air, mechanical philoſophy provides us with ſuitable means; the beſt of which is, perhaps, the common bellows, ſufficiently large, and fitted with leathern tubes.*

That a former contagion, ſome years ago, ſpread from this jail to the town, is a very negative proof in favour of the *nitrous fumigation* nipping the preſent fever in the bud. The cells probably underwent beneficial alterations in the interval: if I miſtake not, in this ſpot Mr. Howard, the champion of humanity, firſt exerciſed his talents. But the difference in the conditions of life, between the victims of the two fevers, might alſo eſſentially aid the means of extinction, or give the infection activity to ſpread further, and ought to be taken into the account. To theſe might be added, the ſeaſon and ſtate of the weather.

Since the ſecond volume of my work was publiſhed, ſeven or eight ſhips are added to our liſt of infections; moſt of the evidence was collected under my own attendance; and a conſiderable part of that offers very new facts. Had thoſe authors who have written on fumigation been on the ſpot, inſtead of reading reports, I am apt to believe, very different concluſions would have been drawn. It is not to be expected that a practice which has prevailed for 3000 years, is to be quickly relinquiſhed; but I do not deſpair of ultimate ſucceſs; *magna eſt veritas et prevalebit.*

I cannot diſmiſs this paper, without adverting to a ſecond alarm ſpread concerning the importation of the plague. Serious indeed! for three ſhips, laden with corn, have been deſtroyed on account of it; and at a period of the utmoſt ſcarcity. It is much to be lamented, that the inquiries which led to this meaſure, had not been publiſhed for the information of the medical world. The peace of ſociety can never be preſerved, if ſuch alarms

* For all purpoſes of ventilation, I would recommend the means of throwing in *pure air* into ſhips, cells, or wards, by mechanical apparatus, if windows and ports are inſufficient, in preference to the negative method of extracting *foul air*.

alarms are to be repeated. Could no advocate for fumigation be found, who would hazard the reputation of the *nitrous vapour*, on the iſſue of a trial that might have ſaved food for fifty thouſand human beings?

> Felix, heu nimium felix! ſi littora tantum,

> Nunquam Dardaniæ tetigiſſent noſtra carinæ.

But this ſubject will occupy more of our attention, ſhould a few years of peace ever enable us to give a more correct and ſyſtematic arrangement of our nautical labours.

---

